# Steering into the Curves: Using Diagnosis to Support the Dignity and Autonomy of Trans Youth

**DOI:** 10.1017/jme.2024.8

**Published:** 2023

**Authors:** Elizabeth R. Boskey, Charlene Galarneau

**Affiliations:** 1: HARVARD MEDICAL SCHOOL, BOSTON, MA, USA.

**Keywords:** Gender-Affirming Care, Trans Youth, Depathologization, Ethics, Respect

## Abstract

This response to Kariyawasam and Rai affirms their critique of the pathologization of trans youth but forecasts a foreseeable negative outcome of their proposed elimination of diagnosis as a prerequisite to gender-affirming care (GAC) — the risk of removing GAC entirely from the medical sphere and compromising the wellbeing of those transgender individuals for whom GAC is deeply affirming. We suggest an ethical framework of GAC that expands past a focus on autonomy to incorporate a principle of respect for persons that affirms the dignity and diversity of trans youth — recognizing the need to facilitate both medical assistance and social change.

In their article, “Taking the long way around: towards a depathologized ethical framework of gender-affirming care for trans youth,”^1^ Kariyawasam and Rai appropriately problematize many of the ways that gender-affirming care (GAC) is currently enacted and advocated for in the US and other Western countries. We, as ethicists, broadly support both their desire to depathologize care and some of their suggestions for doing so. However, depathologization also has a potential for unintended consequences that work against the authors’ intent to transform clinical practice and to expand access to care. The recent bioethics literature is rich with relevant discussions.^2^ Here we call attention to several contextual and ethical considerations that we believe are necessary for a “liberatory praxis of health care for trans youth.”

Pathology is deeply embedded in the biomedical model of health care, functioning in part as a prerequisite for treatment.^3^ This feature of modern medicine is not unique to GAC for trans youth but frames virtually all care within this cultural healing system, which positions as its aim the goal of “saving” or “protecting” persons from more advanced disease and death. The medicalization of common human experiences — pregnancy and childbirth, menopause, dying, short stature, and gender identity — entails pathologization and, frequently, saviorism. Reducing this medical reality to “a harmful ethical shortcut” oversimplifies current and much needed ethical and clinical discussions, especially as the authors acknowledge the elevated risks to mental health of being unable to access GAC.

In the current health care context in the US, and many other countries, it is pathology that enables financial access to treatment. Care that is deemed “medically necessary” is prioritized over care that is perceived as elective — prioritized both in terms of the timing of access and in the way that care is paid for. If there is no pathology, there is no necessity for treatment, thus health insurance becomes unlikely to provide coverage for this care. Removing a diagnosis — whether it be gender dysphoria, gender incongruity, or a diagnosis yet defined — from the paradigm of care makes access to GAC into a luxury rather than a fundamental necessity for the flourishing of some human lives.

Notably, even informed collaborative decision-making care paradigms for GAC involve a diagnosis. The difference is that the diagnosis comes from the medical provider rather than the behavioral health provider and is based in a combination of self-assessment by the person seeking GAC and analysis of the person’s ability to accurately self-assess by the provider. This does not eliminate the diagnosis, as is proposed by the authors, but instead positions the desire for GAC as reflecting a medical need rather than a psychological one. This specific intention, to reformulate the understanding of the desire for medical transition as reflective of an underlying medical condition, was behind the push to redefine the diagnosis of gender identity disorder to gender dysphoria and gender incongruence in official diagnostic classifications.^4^

It is not the diagnosis of gender dysphoria, or gender diversity, that is the core ethical issue. It is the negativity associated with it. What would it be like to combine the ideals of a social model of gender justice with the model of GAC as preventative care that was recently proposed by Arjee Restar? It might involve working to reduce the gendered hostilities and strictures of the world while also recognizing that many individuals who experience gender dysphoria do better when they are supported by medical and other systems. Rather than eliminating gender dysphoria as a diagnosis that allows access to medical treatment, such a model could reduce some of the burdens of diagnosis while still allowing access to its benefits.

Informed consent paradigms are designed to operationalize the ethical principle of respect for autonomy, a respect usually reserved for adults. As such, youth typically are not permitted to give informed consent to medical care except in strictly limited circumstances, which vary by jurisdiction and interpretation. Conversations about the ability of youth to provide informed consent in various circumstances are important and reflect evolving beliefs about the roles and responsibilities of parents and society in the support and protection of children and the ways in which those responsibilities may be culturally and context dependent.

Creating a truly depathologized ethical framework for GAC may be less about removing the diagnosis and more about reconceptualizing it. If the only “pathology” that needs to be addressed is when a person is living in a body whose gendered traits do not allow them to function fully in the world — those discordances are what need to be addressed rather than their underlying gender. In this framework, though discomfort/distress is embedded in persons not society and thus persons not society are “treated,” there is also an implicit recognition that improving society might lessen an individual’s need for care.

Extending ethical discourse about GAC would benefit from an exploration of the principle of respect for persons. Fundamental to the principle of respect for autonomy is an often implied and unexamined recognition of the inherent and equal moral worth of each person, sometimes referred to as human dignity. The principle of respect for persons offers an explicit and capascious appreciation of the whole person beyond simply autonomy, as well as of the diversity of persons, and thus may offer solid justifications for genuinely collaborative decision-making regarding GAC for youth and adults. This would also require, and support, a broader social acknowledgement that being transgender is not a deficit state and is, instead, a facet of diversity to be appreciated and supported.

It is in this diversity-affirming context that the social model of disability becomes most salient to transgender and gender expansive people. The social model of disability appropriately positions accessibility as a problem created by the world and not a deficit experienced by the disabled person. It argues that society should do what it can to make spaces, technology, and all aspects of life available to everyone. The correlary for gender diversity would seem to be working towards removing gendered assumptions from everyday experiences. However, while that would certainly be beneficial, it would not necessarily eliminate many individuals’ need for GAC — even if it could reduce the acuity of discomfort they experience. They would still, therefore, retain a need for a diagnosis, just as individuals with physical and mental disabilities require a diagnosis to access supportive medical care and assistive devices.^5^

It is not the diagnosis of gender dysphoria, or gender diversity, that is the core ethical issue. It is the negativity associated with it. What would it be like to combine the ideals of a social model of gender justice with the model of GAC as preventative care that was recently proposed by Arjee Restar?^6^ It might involve working to reduce the gendered hostilities and strictures of the world while also recognizing that many individuals who experience gender dysphoria do better when they are supported by medical and other systems. Rather than eliminating gender dysphoria as a diagnosis that allows access to medical treatment, such a model could reduce some of the burdens of diagnosis while still allowing access to its benefits.^7^

## Note

The authors have no conflicts to disclose.
